# Trichohyalin-like 1 protein plays a crucial role in proliferation and anti-apoptosis of normal human keratinocytes and squamous cell carcinoma cells

**DOI:** 10.1038/s41420-020-00344-5

**Published:** 2020-10-27

**Authors:** Teruhiko Makino, Megumi Mizawa, Yoko Yoshihisa, Seiji Yamamoto, Yoshiaki Tabuchi, Masashi Miyai, Toshihiko Hibino, Masakiyo Sasahara, Tadamichi Shimizu

**Affiliations:** 1 Department of Dermatology, Faculty of Medicine, Academic Assembly, University of Toyama, Toyama, Toyama, Japan; 2 Department of Pathology, Faculty of Medicine, Academic Assembly, University of Toyama, Toyama, Toyama, Japan; 3 Division of Molecular Genetics Research, Life Science Center, University of Toyama, Toyama, Toyama, Japan; 4 Shiseido Global Innovation Center, Yokohama, Kanagawa Japan

**Keywords:** Cell growth, Cell death, Cell signalling, Skin cancer

## Abstract

Epidermal differentiation is a complex process that requires the regulated and sequential expression of various genes. Most fused-type S100 proteins are expressed in the granular layer and it is hypothesized that these proteins may be associated with cornification and barrier formation. We previously identified a member of the fused-type S100 proteins, Trichohyalin-like 1 (TCHHL1) protein. TCHHL1 is distributed in the basal layer of the normal epidermis. Furthermore, the expression is markedly increased in cancerous/non-cancerous skin samples with the hyperproliferation of keratinocytes. We herein examined the role of TCHHL1 in normal human keratinocytes (NHKs) and squamous cell carcinoma (SCC). The knockdown of TCHHL1 by transfection with TCHHL1 siRNA significantly inhibited proliferation and induced the early apoptosis of NHKs. In TCHHL1-knockdown NHKs, the level of extracellular signal-regulated kinase 1/2 (ERK1/2) phosphorylation was markedly decreased. In addition, the slight inhibition of v-akt murine thymoma viral oncogene homolog (AKT) phosphorylation and upregulation of forkhead box-containing protein O1(FOXO1), B-cell lymphoma2 (BCL2) and Bcl2-like protein 11 (BCL2L11) was observed. Skin-equivalent models built by TCHHL1-knockdown NHKs showed a markedly hypoplastic epidermis. These findings highlight that TCHHL1 plays an important role in homeostasis of the normal epidermis. TCHHL1 was expressed in the growing cells of cutaneous SCC; therefore, we next examined an association with the cell growth in HSC-1 cells (a human SCC line). In HSC-1 cells, the knockdown of TCHHL1 also suppressed cell proliferation and induced apoptosis. These cells showed an inhibition of phosphorylation of ERK1/2, AKT and signal transducers and activator of transcription 3, and the significant upregulation of FOXO1, BCL2, and BCL2L11. Accordingly, TCHHL1 is associated with survival of cutaneous SCC. In addition, we hypothesize that TCHHL1 may be a novel therapeutic target in cutaneous SCC.

## Introduction

Epidermal differentiation is a complex process that requires the regulated and sequential expression of various genes. Several genes involved in this process are found within a 2 Mb region at chromosome band 1q21.3 called the epidermal differentiation complex^1,2^. A family of proteins described as “fused S100 proteins” containing the EF-hand domain in the N-terminus followed by multiple tandem peptide repeats is located in this region^3–8^. Most fused-type S100 proteins, such as filaggrin, are expressed in the granular layer and it is hypothesized that these proteins may be associated with the cornification or barrier formation. The findings that mutation of the *filaggrin* gene causes ichthyosis vulgaris^9^ or atopic dermatitis^10^ and that the expression of filaggrin-like proteins is decreased in atopic skin^11^ may support this hypothesis.

Trichohyalin-like 1 (TCHHL1) is a member of the fused S100 proteins, which was recently identified (GenBank accession number: AY456639.1)^12^. The human *TCHHL1* gene encodes a protein of 904 amino acids and the deduced amino acid sequence contains an EF-hand domain in the N-terminus followed by a large domain. TCHHL1 is expressed in the basal layer of the normal epidermis. The expression of TCHHL1 was increased in skin samples with the hyperproliferation of keratinocytes, such as psoriasis vulgaris, basal cell carcinoma, and squamous cell carcinoma (SCC)^12^. In addition, the expression of TCHHL1 was enhanced in the proliferating keratinocytes of the epidermis damaged by ultraviolet irradiation^13^. These findings suggest that TCHHL1 is associated with the proliferation of keratinocytes.

In the present study, we investigated the role of TCHHL1 in normal human keratinocytes (NHKs) and SCC cells. Consequently, the results of the present study showed that TCHHL1 is associated with proliferation and anti-apoptosis of NHKs and SCC cells via the activation of ERK1/2 or AKT, and that TCHHL1 therefore plays an important role in homeostasis of the normal epidermis and the survival of cutaneous SCC.

## Results

### The knockdown of TCHHL1 inhibits the cell growth of NHKs

**Fig. 1:**
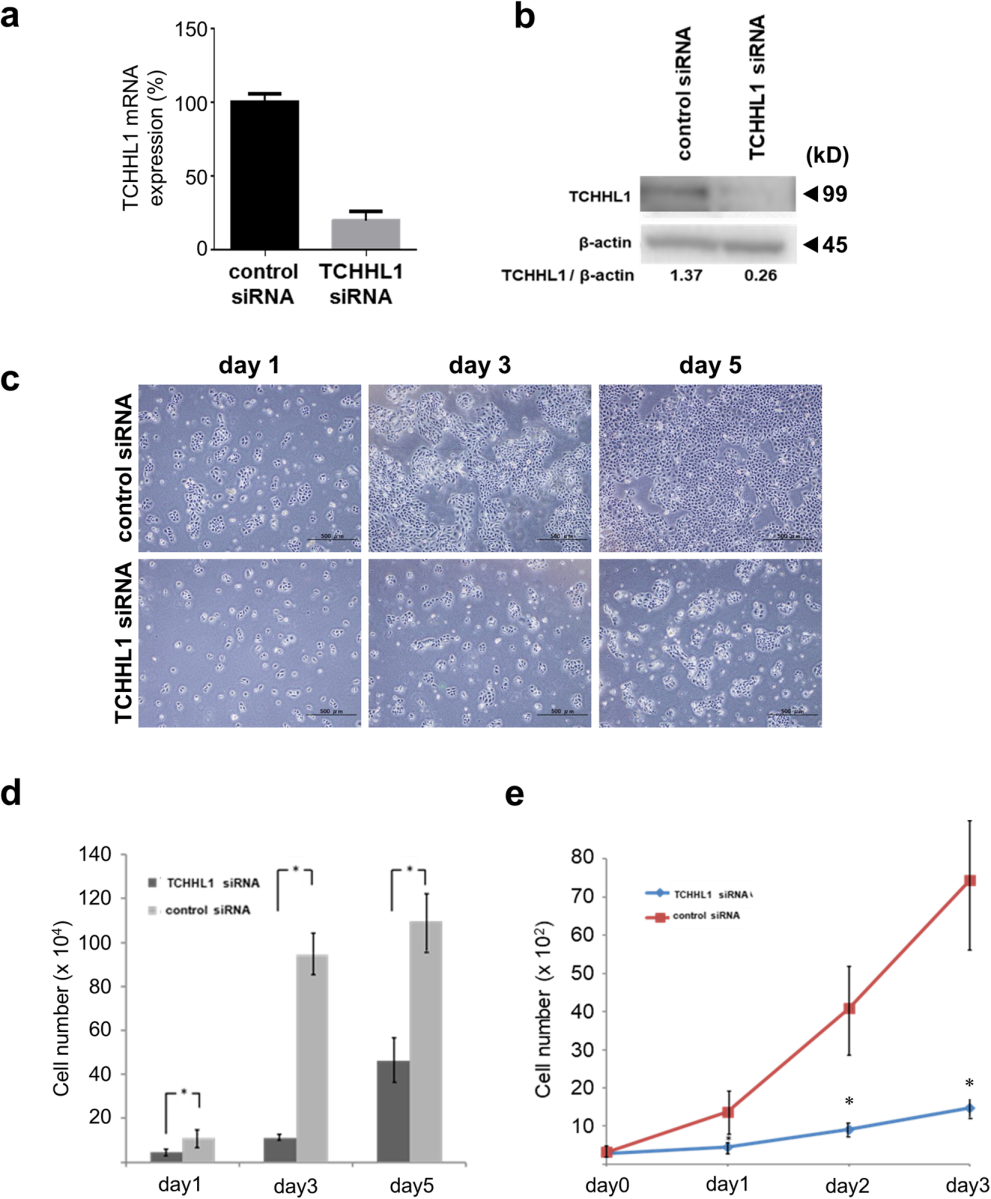
The knockdown of TCHHL1 inhibits cell growth in normal human keratinocytes. **a** TCHHL1 siRNA#1 strongly suppressed the expression of TCHHL1 mRNA to 17.1%. **b** Immunoblotting showed a marked reduction of TCHHL1 protein (19%). Full-length blots are presented in Supplementary Fig. S1b. **c** Representative micrographs of growing NHKs at 1, 3, and 5 days after transfection of TCHHL1 siRNA or control siRNA. **d** Quantification of the number of growing NHKs at 1, 3, and 5 days after transfection with TCHHL1 siRNA or control siRNA. The data represent the mean ± SD of three independent experiments. *p < 0.05. **e** The knockdown of TCHHL1 due to TCHHL1 siRNA#1 suppresses growth rate of NHKs, determined by MTS assay. The data represent the mean ± SD of three independent experiments. **p < 0.01.

To investigate the effect of TCHHL1 in NHKs, we performed siRNA experiments using TCHHL1 siRNA. The TCHHL1 siRNA#1 (s43057) treatment efficiently suppressed the mRNA and protein expressions of TCHHL1 to 17.1% and 19.0% of the control levels, respectively (Fig. 1a, b). The TCHHL1#2 siRNA (s43059) also suppressed the mRNA expressions of TCHHL1 to 22.5% (Fig. S1a, b). The cell viability of NHKs, which were transfected with TCHHL1 siRNAs, was assessed at 1, 3, or 5 days after transfection. From one day after transfection, cultures of TCHHL1-knockdown NHKs showed significantly reduced numbers of viable cells in comparison to control NHKs (Figs. 1c, d and S1c). MTS assay also demonstrated that TCHHL1-knockdown NHKs significantly decreased the growth rate in comparison to control NHKs (Figs. 1e and S1d). The two kinds of TCHHL1 siRNAs showed an equal effect in NHKs. We therefore decided to use TCHHL1 siRNA#1 for the further experiments.

### TCHHL1 is associated with the proliferation and apoptosis of NHKs

**Fig. 2:**
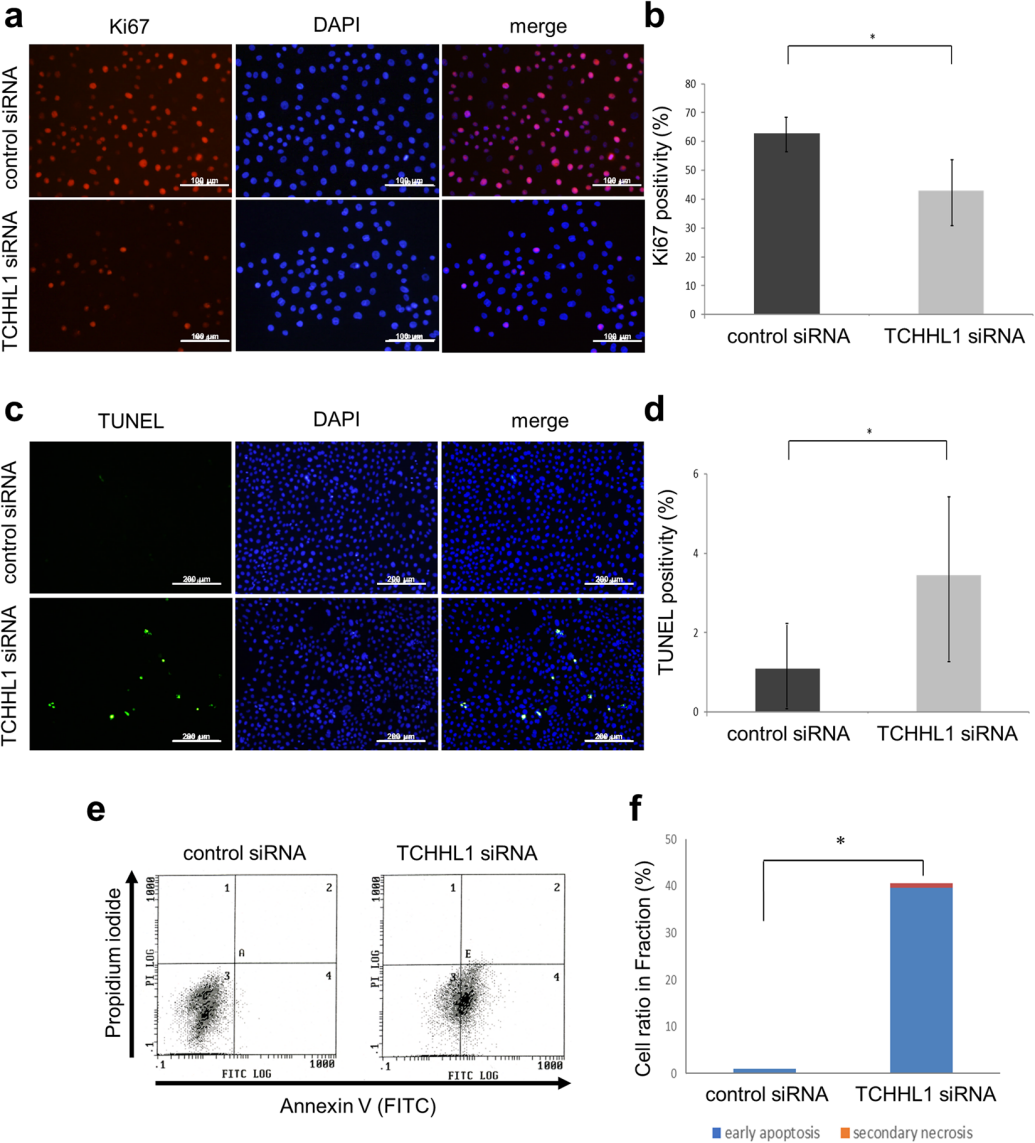
TCHHL1 is associated with the proliferation and apoptosis of normal human keratinocytes. **a** TCHHL1 siRNA or control siRNA were transfected into growing NHKs that were stained with an anti-Ki67 antibody (left panels). Nuclear staining by 6-diamidine-2′-phenylindole dihydrochloride (DAPI) appears in blue. Merged figures with nuclear staining are also shown (right panels). **b** The quantification of Ki67-positive cells after transfection of TCHHL1 siRNA or control siRNA. In each case, the positive cells in 10 fields were counted and summed. The data represent the mean ± SD of three independent experiments. *p < 0.01. **c** Growing NHKs transfected with TCHHL1 siRNA or control siRNA were subjected to TUNEL staining (left panels). Nuclear staining with DAPI appears in blue. Merged figures with nuclear staining are also shown (right panels). **d** Quantification of TUNEL-positive cells after transfection of TCHHL1 siRNA or control siRNA. In each case, the positive cells in 10 fields were counted and summed. The data represent the mean ± SD of three independent experiments. *p < 0.01. **e** Growing NHKs, which were transfected with TCHHL1 siRNA or control siRNA, were stained with annexin V and PI. The cells were assessed by a FACS analysis at 3 days after transfection of siRNAs. **f** The percentage of early apoptotic cells (annexin V-positive and PI-negative cells; TCHHL1 siRNNA: 56.4%, control siRNA: 1.04%) and secondary necrotic cells (annexin V-positive and PI-positive cells; TCHHL1 siRNNA: 1.06%, control siRNA: 0%) is shown. The data represent the mean ± SD of three independent experiments. **p < 0.05.

We next examine the effect of TCHHL1 in the proliferation or apoptosis of NHKs. The number of Ki67-positive cells significantly decreased in TCHHL1-knockdown NHKs in comparison to that in control NHKs (Fig. 2a, b). In contrast, the number of TUNEL-positive cells significantly increased in TCHHL1-knockdown NHKs in comparison to that of control NHKs (Fig. 2c, d). Furthermore, to clarify the effect of TCHHL in apoptosis, an annexin V-PI analysis was performed. Annexin V staining revealed a significant increase in early apoptotic cells (56.4%) on day 3 after the transfection of TCHHL1 siRNA, in comparison to control siRNA (1.06%) (Fig. 2e, f).

### The analyses of the gene expression, biological function, and gene network in TCHHL1-knockdown NHKs

**Fig. 3:**
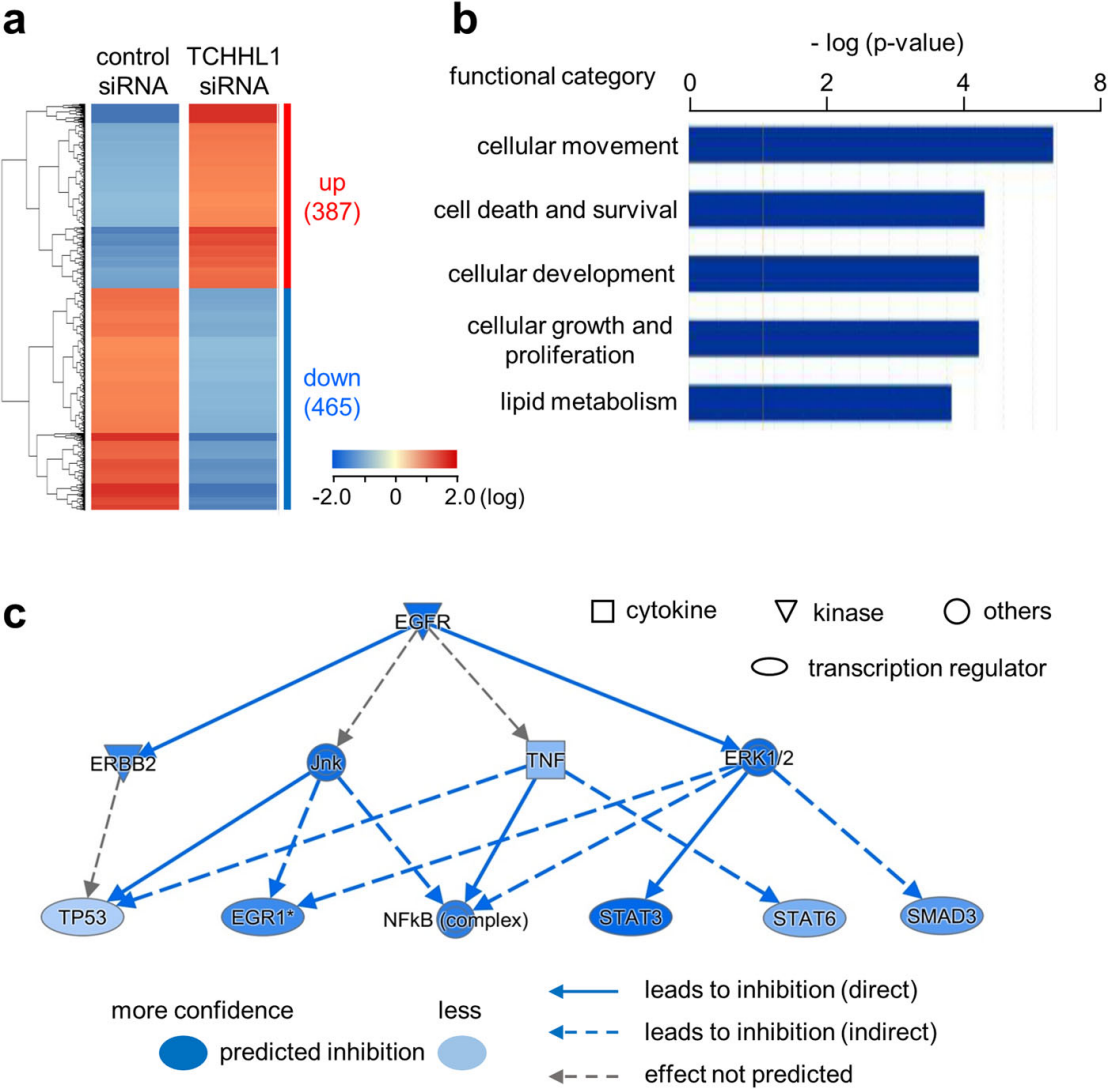
Analyses of gene expression, biological function, and gene network in TCHHL1-knockdown NHKs. **a** Hierarchical clustering of 852 probe sets differentially expressed by >2.0 in the in NHKs transfected with TCHHL1 siRNA or control siRNA. Clustering was carried out using the GeneSpring® GX software program. The number in parentheses indicates the number of probe sets. **b** The functional category analysis. A total of 313 upregulated and 367 downregulated, functionally annotated genes were identified using the Ingenuity® Pathway Analysis tools. The top five biological functions are shown. **c** The mechanistic gene network. Differentially expressed genes were analyzed by the Ingenuity® Pathway Analysis tools. The mechanistic gene network is graphically displayed as nodes (genes or proteins) and edges (the biological relationships between the nodes). Nodes and edges are displayed by various shapes and labels that represent the functional classes of genes and the nature of the relationship between nodes, respectively.

To examine the gene expression changes associated with TCHHL1 in NHKs, we performed gene expression profiling on NHKs treated with either TCHHL1 siRNA or control siRNA. Of the 54,675 probe sets analyzed, 17,369 probe sets were significantly expressed in either TCHHL1 siRNA or control siRNA-treated NHKs. A GeneSpring^®^ software-based expression analysis demonstrated that many probe sets that were differentially regulated by a factor of ≥2.0. As shown in a hierarchical clustering heatmap (Fig. 3a), 387 and 465 probe sets were found to be upregulated and downregulated, respectively, in the cells treated with siRNA for TCHHL1. In addition, biological function and gene network analyses were conducted using the Ingenuity^®^ Pathways Knowledge Base. A total of 313 upregulated and 367 downregulated, functionally annotated genes were identified in the 387 and 465 differentially expressed probe sets, respectively. The top five biological functions including “cellular movement”, “cell death and survival”, and “cellular development” in all annotated genes are shown in Fig. 3b. Interestingly, in a functional category analysis, apoptosis in the category of “cell death and survival” was predicted to be an activation state (activation *z*-score: 2.309). Many pro-apoptic and anti-apoptotic genes were upregulated and downregulated in NHKs treated with TCHHL1 siRNA, respectively (Figs. S2, S3). An upstream analysis also demonstrated that the activities of transcription factors, including TP53, early growth response-1, nuclear factor-kappa B, STAT3, STAT6, and SMAD3, were regulated by upstream signaling molecules, including Erb-B2 Receptor Tyrosine Kinase 2, c-jun N-terminal kinase, tumor necrosis factor and ERK1/2, and the regulation of intermediate signaling molecules converged on a potential master regulator, EGFR (Fig. 3c).

### TCHHL1 affects the activation of ERK1/2 and AKT in NHKs

**Fig. 4:**
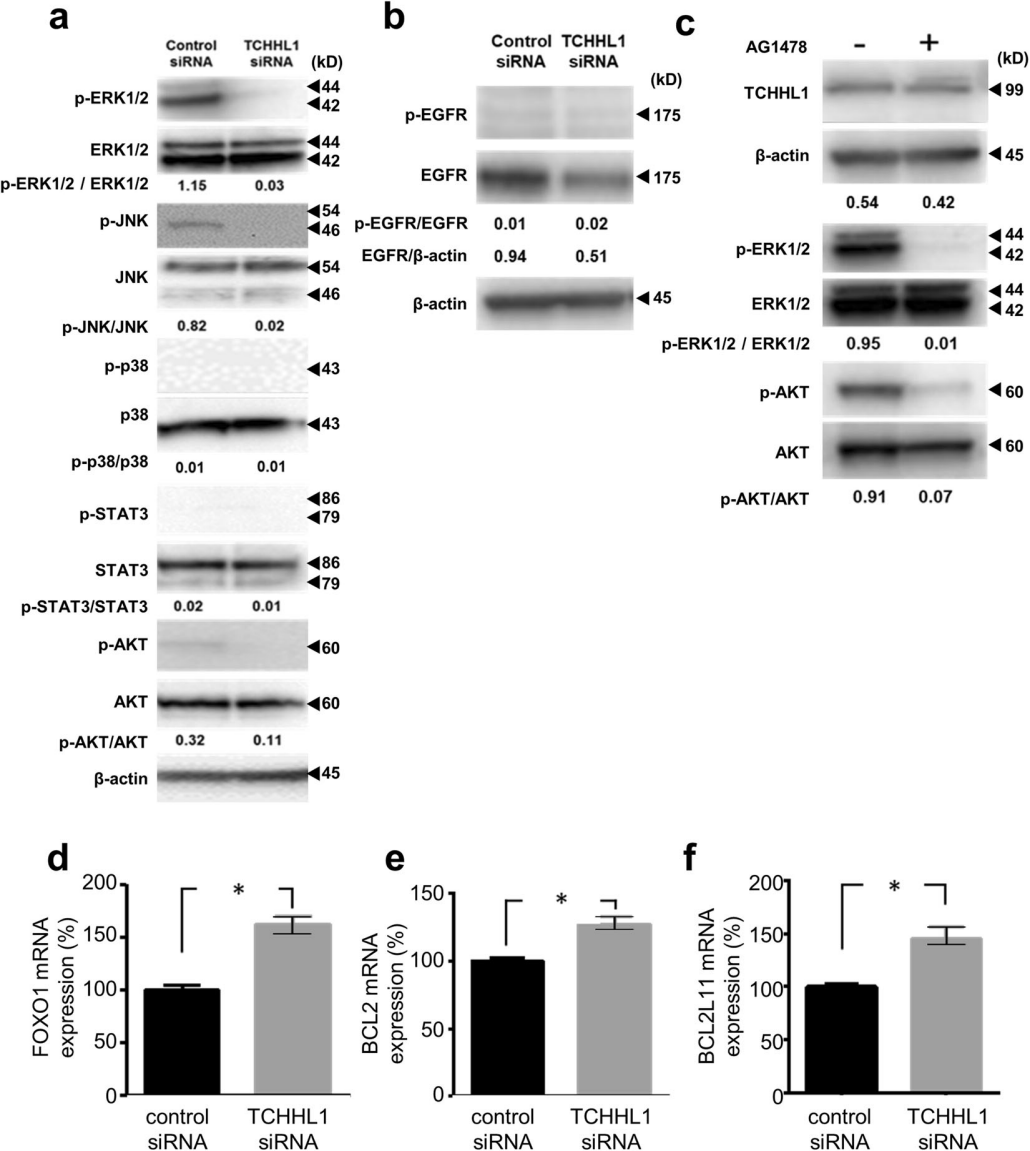
TCHHL1 is associated with cellular proliferation via the activation of mitogen-activated protein kinase. **a** Western blotting of NHKs transfected with TCHHL1 siRNA or control siRNA. Full-length blots are presented in Supplementary Fig. S4. **b** Western blotting of NHKs transfected with TCHHL1 siRNA or control siRNA. Full-length blots are presented in Supplementary Fig. S5. **c** Western blotting of NHKs treated with EGFR inhibitor, AG1478. Full-length blots are presented in Supplementary Fig. S6. **a**–**c** β-actin was used as a loading control. Band densities were measured using the Image J software program. The data shown are representative of three independent experiments. **d**–**f** Relative mRNAs in NHKs with TCHHL1 siRNA or control siRNA were analyzed by quantitative RT-PCR, normalized to the β-actin value. All data represent the mean ± SD of three independent experiments. *p < 0.01.

To clarify the underlying mechanism through which TCHHL1 is associated with cell growth, we examined the effects of TCHHL1 in the MAPK and AKT signal pathways. The level of ERK1/2 phosphorylation markedly decreased in TCHHL1-knockdown NHKs in comparison to those of control NHKs (Figs. 4a and S4). Phosphorylation of SAPK/JNK and AKT, which was slightly detected in control NHKs, was completely disappeared in TCHHL1-knockdown NHKs. Decreased expression of EGFR was observed in TCHHL1-knockdown NHKs; however, phosphorylation of EGFR was comparable in both TCHHL1-knockdown and control NHKs (Figs. 4b and S5). In addition, the treatment of NHKs with an EGFR inhibitor (AG1478) markedly suppressed the phosphorylation of ERK1/2 and AKT; however, there was no effect on the TCHHL1 expression (Figs. 4c and S6). We also examined the association between the KLF4 and TCHHL1 expression because TCHHL1 was recently reported to be a target gene of KLF4, which is a member of the Kruppel-like family of transcription factors^14^. The expression of TCHHL1 was significantly suppressed in the KLF4-knockdown NHKs (Fig. S7). We furthermore examined an expression level of apoptosis-associated molecules, which were existed in down-stream of AKT signal pathway because the phosphorylation of AKT was suppressed in TCHHL1-knockdown NHKs. Consequently, the expression levels of forkhead box-containing protein O1 (FOXO1) (Fig. 4d), B-cell lymphoma2 (BCL2) (Fig. 4e) and Bcl2-like protein 11 (BCL2L11) (Fig. 4f) significantly increased in TCHHL1-knockdown NHKs.

### TCHHL-knockdown-induced hypoplastic epidermal tissue

**Fig. 5:**
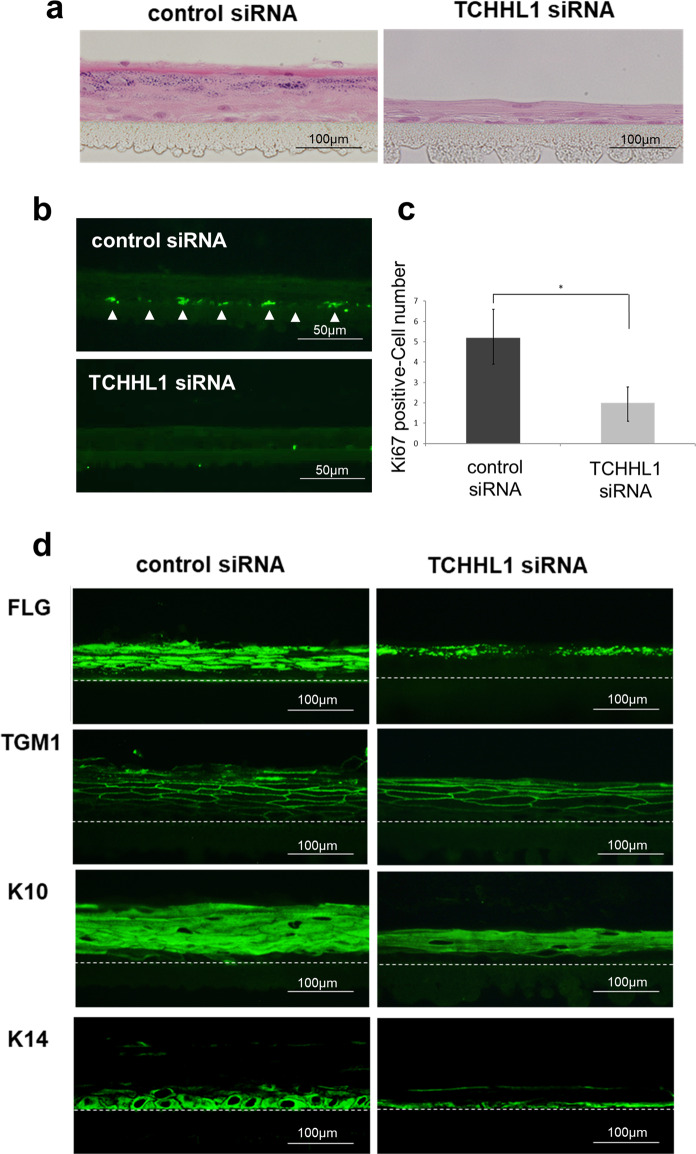
TCHHL1 depletion inhibits proliferation in human epidermal tissue. **a** Skin-equivalent models were prepared using NHKs transfected with TCHHL1 siRNA or control siRNA. A Hematoxylin–Eosin-stained image is shown. Scale bar: 50 μm. **b** Skin equivalent models treated TCHHL1 siRNA or control siRNA were stained with anti-Ki67 antibody. The arrowheads indicate Ki67-positive cells. Scale bar: 100 μm. **c** A quantitative analysis of the Ki67-positive cells in the basal layer. Ki67-positive cells were counted in each 500-μm field (total length 5.0 mm) and expressed as the mean ± SD of three independent experiments. *p < 0.01. **d** Skin-equivalent models treated with TCHHL1 siRNA or control siRNA were stained with antibodies against filaggrin (FLG), transglutaminase 1 (TGM1), keratin 10 (K10), and keratin 14 (K14). The dotted line indicates the dermo-epidermal junction. Scale bar: 50 μm.

To examine the effect of TCHHL1 on the skin tissue construction, we built skin-equivalent models using TCHHL1 siRNA-transfected NHKs. TCHHL1-knockdown-skin-equivalent models showed a markedly hypoplastic epidermis and the horny and granular layers were hardly observed (Fig. 5a). The numbers of Ki67-positive cells in TCHHL1-knockdown epidermis were significantly decreased in comparison to the control epidermis (Fig. 5b, c). In contrast, the TCHHL1-knockdown epidermis maintained the expression of differentiation markers, including filaggrin, transglutaminase1, and keratin 10 although the expression of keratin 14 decreased (Fig. 5d).

### The expression of TCHHL1 in cutaneous SCC

**Fig. 6:**
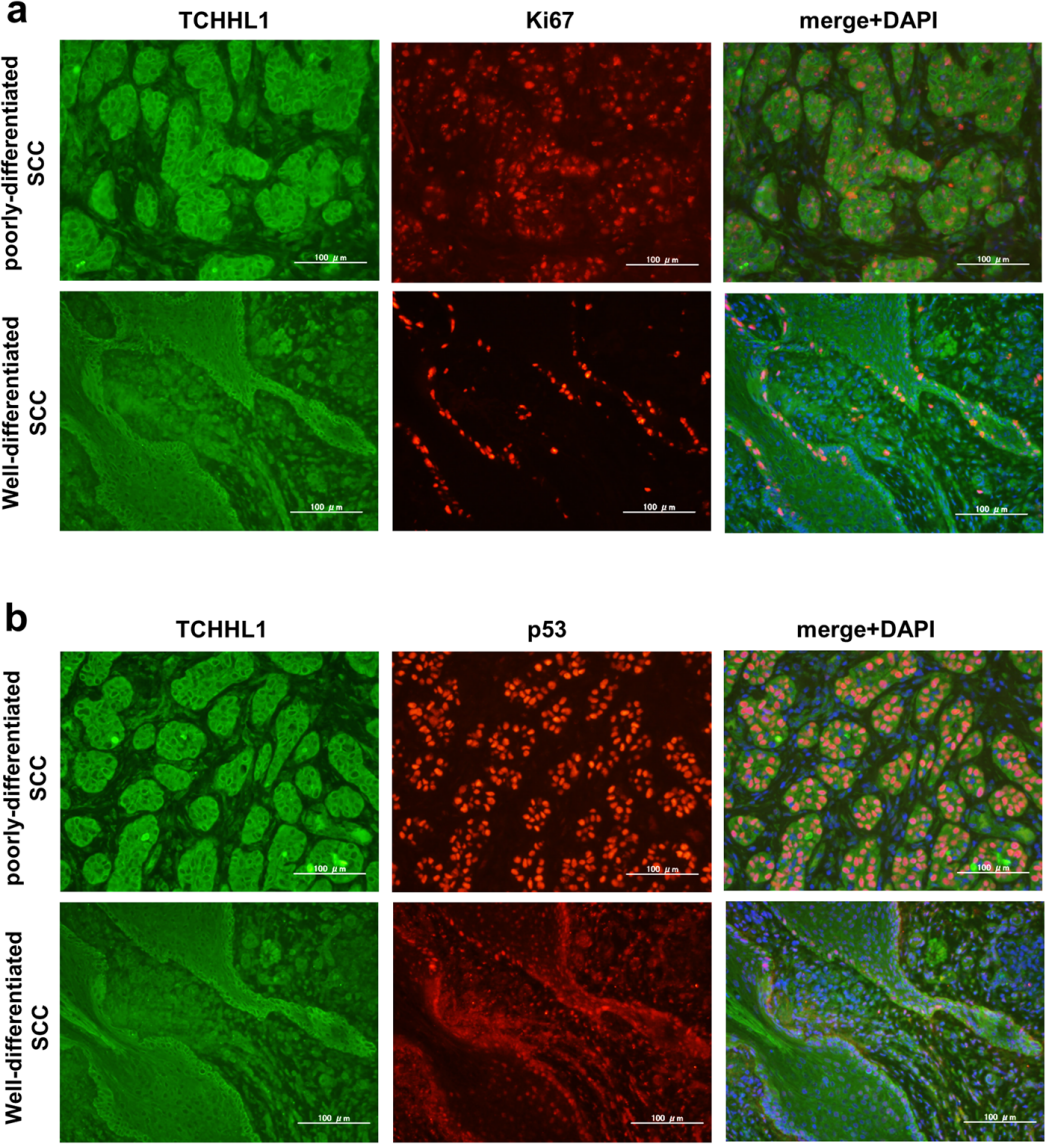
TCHHL1 is expressed in the proliferative cell of cutaneous squamous cell carcinomas. Tissue specimens obtained from cutaneous SCCs (low-differentiated type and high-differentiated type) were double-immunostained for TCHHL1 and Ki67 **a** or for TCHHL1 and p53 **b**. Scale bars: 100 μm (in all panels).

To clarify an association of TCHHL1 with the growth of cutaneous SCC, we examined the expression of TCHHL1 using tissue specimens obtained from either poorly differentiated or well-differentiated cutaneous SCCs (Fig. S8). In the poorly differentiated-SCC samples, the tumor nests were relatively small and almost all tumor cells strongly expressed TCHHL1. The signals of Ki67 or p53 were also observed in these tumor cells (Fig. 6a, b upper panels). In contrast, in well-differentiated SCCs, the signals of TCHHL1 were observed to be stronger in the peripheral areas than in the center areas of the tumor nests. Ki67-positive or p53-positive cells were distributed within the TCHHL1-positive area of the tumor nests (Fig. 6a, b lower panels).

### Knockdown of TCHHL1 suppresses the growth of SCC cells

**Fig. 7:**
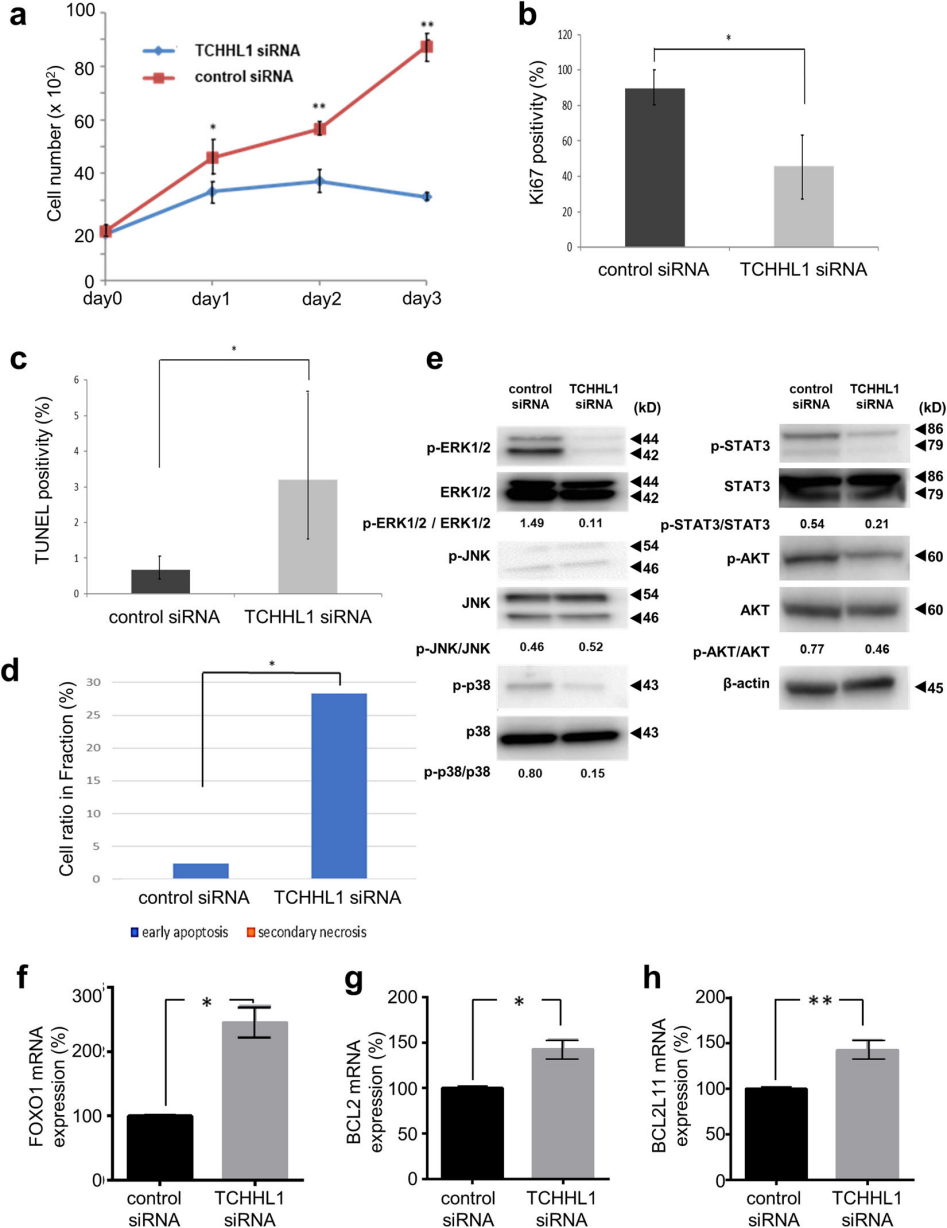
TCHHL1 knockdown suppresses the proliferation of squamous cell carcinoma cells. **a** TCHHL1 knockdown with TCHHL1 siRNA suppressed the growth rate of HSC1, as determined by an MTS assay. The data represent the mean ± SD of three independent experiments. *p < 0.05, **p < 0.01. **b** The quantification of Ki67-positive HSC1 cells after transfection of TCHHL1 siRNA or control siRNA. In each case, the positive cells in 10 fields were counted and summed. The data represent the mean ± SD of three independent experiments. *p < 0.01. **c** The quantification of TUNEL-positive HSC1 cells after the transfection of TCHHL1 siRNA or control siRNA. In each case, the positive cells in 10 fields were counted and summed. The data represent the mean ± SD of three independent experiments. *p < 0.01. **d** The percentage of early apoptotic cells (TCHHL1 siRNNA: 28.3%, control siRNA: 2.42%) and secondary necrotic cells (TCHHL1 siRNNA: 0.05%, control siRNA: 0%) is shown. The data represent the mean ± SD of three independent experiments. *p < 0.05. **e** Immunoblotting of HSC-1 cells transfected with TCHHL1 siRNA or control siRNA. β-actin was used as a loading control. Band densities were measured using the Image J software program. Full-length blots are presented in Supplementary Fig. S11. The data shown are representative of three independent experiments. **f**–**h** Relative mRNAs in HSC-1 cells with TCHHL1 siRNA or control siRNA were studied by quantitative RT-PCR, normalized to the β-actin value. All data represent the mean ± SD of three independent experiments. *p < 0.01.

To clarify the effect of TCHHL1 on the growth of SCC, we performed siRNA experiments using HSC-1 cell (a cutaneous SCC cell line)^15^. In HSC-1 cells, TCHHL1 siRNA treatment efficiently knocked down the mRNA and protein expression of TCHHL1 to 12.1% and 21.2% of the control levels, respectively (Fig. S9). Next, the cell viability of HSC-1 cells, which were treated with TCHHL1 siRNA, was assessed at 1, 3, and 5 days after transfection. Consequently, cultures of TCHHL1-knockdown HSC-1 cells showed significantly reduced number of viable cells from 3 days after transfection (Fig. S10a, b). In the MTS assay, TCHHL1-knockdown HSC-1 cells showed a significantly decreased cell growth rate (Fig. 7a). In TCHHL1-knockdown HSC-1 cells, the number of Ki67-positive cells was significantly decreased (Figs. 7b and S10c) and the number of TUNEL-positive cells was significantly increased (Figs. 7c and S10d). An annexin V-PI analysis showed an increase of early apoptotic cells (28.3%) at day 3 after transfection of TCHHL1 siRNA in comparison to control siRNA (2.42%) (Figs. 7d and S10e). The level of ERK1/2 phosphorylation was markedly inhibited in TCHHL1-knockdown HSC-1 cells (Figs. 7e and S11). The phosphorylation of p38 MAPK, STAT3, or AKT was suppressed by the knockdown of TCHHL1 in HSC-1 cells. We finally examined the expression level of apoptosis-associated molecules. Consequently, the expression levels of FOXO1 (Fig. 7f), BCL2 (Fig. 7g), and BCL2L11 (Fig. 7h) in TCHHL1-knockdown HSC-1 cells were significantly increased in comparison to control HSC-1 cells.

## Discussion

TCHHL1 was previously reported to be expressed in the basal layer of the normal epidermis and the increased expression of TCHHL1 was observed in skin disorders with hyperproliferative keratinocytes^12^. These findings suggested an association between TCHHL1 and the proliferation of keratinocytes. The present study demonstrated that the knockdown of TCHHL1 inhibited the proliferation of NHKs and TCHHL1-knockdown NHKs showed a decrease in the phosphorylation of the mitogen-activated protein kinases (MAPKs), especially ERK1/2. Furthermore, the skin-equivalent model constructed by TCHHL1-knockdown NHKs showed marked hypoplastic epidermis. ERK1/2 is primarily activated by mitogenic stimuli and mediates cell growth^16,17^, and ERK1/2 signaling promotes the proliferation of basal keratinocytes and suppresses normal differentiation to suprabasal keratinocytes in the normal epidermis^18^. Furthermore, the activation of ERK1/2 was reported to be upregulated in psoriatic skins, which shows hyperproliferation of keratinocytes^19^. In contrast, the double-knockdown of ERK1 and ERK2-induced hypoplasia of the epidermis without disrupting differentiation in a skin-equivalent model^20^. This phenotype well resembled that of the skin-equivalent model lacking TCHHL1. These findings of the present study suggest that TCHHL1 affects the proliferation of NHKs via the activation of ERK1/2.

Interestingly, the knockdown of TCHHL1 also induced early apoptosis in NHKs. In TCHHL1-knockout NHKs, the inhibition of AKT phosphorylation and the significant increase of apoptosis-related molecules, including FOXO1, BCL2, and BCL2L11, were observed. The PI3K/AKT signal pathway is also a key regulator of proliferation, survival, migration, and angiogenesis in human cells^21^. The inhibition of the PI3K/AKT signal pathway is widely known to induce cell apoptosis^21^. In addition, AKT regulates caspase activity in the initiation and progression of the differentiation process of NHKs, which is an altered form of apoptosis^22^. Accordingly, the present study demonstrated that TCHHL1 is associated with not only proliferation but also the anti-apoptosis of NHKs via the activation of the AKT signaling pathway.

The activation of EGFR is known to lead to the recruitment and phosphorylation of several downstream substrates, including ERK1/2 and Phosphoinositide 3 kinase (PI3K)^23^. The knockdown of TCHHL1 showed no effect for phosphorylation of EGFR although it induced a slight reduction of the EGFR expression. In addition, the inhibition of phosphorylation of EGFR by AG1478 did not affect the TCHHL1 expression level. In contrast, the knockdown of KLF4, which is also known to regulate the proliferation and differentiation of cells^14^, significantly reduced the expression level of TCHHL1. We therefore hypothesize that the activation of ERK1/2 or AKT by TCHHL1 seems to be independent of EGFR phosphorylation. In addition, the expression of TCHHL1 may be regulated by KLF4 not by EGF/EGFR signaling; however, further research will be required to clarify this.

Cutaneous SCC is a malignant neoplasm derived from epidermal keratinocytes and is the most common type of skin cancer. The present study demonstrated the strong expression of TCHHL1 in tumor cells of cutaneous SCCs and at sites that corresponded with the distribution of the ki67-positive or p53-positive cells. Furthermore, the knockdown of TCHHL1 inhibited the phosphorylation of ERK1/2, STAT3, and AKT and significantly increased the expression of FOXO1, BCL2, and BCL2L11, resulting in the inhibition of proliferation and the induction of apoptosis in HSC-1 cell. Recent studies demonstrated that the tumor cells of SCC had abnormalities in signal transduction pathways, such as transforming growth factor-β/Smad, mammalian target of rapamycin complex 1 or JNK^24–26^. The activation of ERK1/2, STAT3, and AKT was also reported in cutaneous SCC and HSC-1 cells^27,28^. The activation of STAT3 though EGFR or PI3K/AKT activation plays a crucial role in the proliferation and survival of cutaneous SCC^29^. ERK1/2 activation may also be associated with the development of cutaneous SCC because vemurafenib, a BRAF inhibitor, can induce cutaneous SCC as an adverse effect via the trans-activation of downstream ERK signaling^30^. Therefore, TCHHL1 also affect the proliferation and survival of cutaneous SCC through the activation of ERL1/2, AKT, and STAT3 signal pathways. In addition, we hypothesize that the inhibition of TCHHL1 may be a novel therapeutic option for cutaneous SCC.

Taken together, the present study showed that TCHHL1 plays an important role in the homeostasis of the normal epidermis and the growth of cutaneous SCC. Further research will be required to clarify the precise mechanism through which the TCHHL1 expression is regulated, the relationship between TCHHL1 and other molecules, and the possible application of TCHHL1 as a therapeutic target for cutaneous SCC; however, the present study helps to expand knowledge on the roles of TCHHL1 in the skin and SCC.

## Methods and materials

### Clinical materials

Tissue samples from patients with poorly differentiated (*n* = 5; mean age, 79.8 years) or well-differentiated (*n* = 5; mean age, 77.2 years) cutaneous SCC were obtained from Toyama University Hospital. The diagnosis of SCC was made based on histological findings by experienced dermatologists and pathologists. All patients gave their written informed consent and the study protocol complied with all of the Principles of the Declaration of Helsinki. This study was approved by the Medical Ethics Committees of the University of Toyama, Toyama, Japan (approval number: 29-139).

### Cells and cell culture

NHKs (Kurabo Industries Ltd, Osaka, Japan) and the human SCC line, HSC-1 cells (Japanese Collection of Research Bioresources Cell Bank, Osaka, Japan) were cultured in Humedia-KG2 (Kurabo Industries Ltd) and Dulbecco’s modified Eagle medium (Nacalai Tesque, Kyoto, Japan) supplemented with 20% fetal bovine serum (Sigma-Aldrich, St. Louis, MO) at 37 °C in a humidified atmosphere under 5% CO_2_. All experiments were carried out on cells at the third passage.

### Reagents and antibodies

Pre-designed small interfering RNA (siRNA) directed against human TCHHL1 (s43057 and s43059) and Kruppel-like factor 4 (KLF4; s17795) and negative control siRNA were purchased from Life Technologies (Carlsbad, CA). An epidermal growth factor receptor (EGFR) inhibitor (AG 1478) was purchased from Abcam (Cambridge, UK). An antibody against an oligopeptide (HPQRERLVLQREASTTKQ) corresponding to part of the C-terminal region of TCHHL1 was previously generated^12^. Antibodies against extracellular signal-regulated kinase 1/2 (ERK1/2; #4695), phospho-ERK1/2 (#4370), stress-activated protein kinase/c-jun N-terminal kinase (SAPK/JNK; #9252), phospho-SAPK/JNK (#4668), p38 mitogen-activated protein kinase (p38 MAPK; #8690), phospho-p38 MAPK (#4511), v-akt murine thymoma viral oncogene homolog (AKT; #9272), phospho-AKT (#9271), signal transducers and activator of transcription 3 (STAT3; #9132), phospho-STAT3 (#9145), EGFR(#4267), phospho-EGFR (#4407) and β-Actin (#4967) were purchased from Cell Signaling Technology Japan, K.K. (Tokyo, Japan). Antibodies against cytokeratin-10 (M7002), Ki67 (M7240), and p53 (M7001) were purchased from DAKO (Carpinteria, CA). Antibodies against human cytokeratin-14 (ab7800) and filaggrin (ab218863), and antibodies against transglutaminase1 (PA5-59088) were purchased from Abcam and Thermo Fisher Scientific Inc. (Waltham, MA), respectively.

### RNA interference

For transient knockdown, NHKs or HSC-1 cells were transfected with siRNA duplexes using Lipofectoamine RNAiMAX (Thermo Fisher Scientific Inc.) according to the manufacturer’s instructions. At 3 days after transfection, the cells were collected and used for the experiments.

### RNA preparation

Total RNA was isolated from cells using a NucleoSpin® RNA Plus isolation kit (MARCHEREY-NAGEL GmbH & Co., Duren, Germany) and treated with Nucleospin genomic DNA removal column (MARCHEREY-NAGEL GmbH & Co.) to remove residual genomic DNA. The concentration of RNA was measured by spectroscopy with an expected A260/A280 ratio close to 2. Qualitative assessment of the RNA was also checked using a Bioanalyzer 2100 (Agilent Technologies, Inc., Santa Clara, CA). RNA samples (RNA integrity number values: >9.5) were used.

### Quantitative reverse transcription polymerase chain reaction (RT-PCR)

All RNA samples were confirmed to give no positive signals without reverse transcription. Reverse transcription was performed with random hexamers and Superscript III (Life Technologies). Complementary DNA samples were analyzed using an SYBR Premix Ex Taq™ II (Takara Bio Inc., Shiga, Japan) according to the manufacturer’s instructions. All experiments were performed in triplicate and the β-actin levels were normalized. The primers used quantitative RT-PCR are shown in Table 1.

**Table 1:** Primers for quantitative RT-PCR.

TCHHL1	FW: AATTAAAGGTCCAAGGCCCAAG
	RV: GGAGGCTGAATTGTCCTCATCTA
KLF4	FW: AAGAGTTCCCATCTCAAGGCACA
	RV: GGGCGAATTTCCATCCACAG
FOXO1	FW: GATGGTCAAGAGCGTGCCCTA
	RV: TGGATTGAGCATCCACCAAGAA
BCL2	FW: AACATCGCCCTGTGGATGAC
	RV: AGAGTCTTCAGAGACAGCCAGGAG
BCL2L11	FW: CATCATCGCGGTATTCGGTTC
	RV: AAGGTTGCTTTGCCATTTGGTC
ACTB	FW: TGGCACCCAGCACAATGAA
	RV: CTAAGTCATAGTCCGCCTAGAAGCA

### Preparation of skin-equivalent models

NHKs, which were transfected with TCHHL1 siRNA or control siRNA, were plated in chambers (CELLnTEC, Bern, Switzerland) at 1 day after transfection. Two days later, the cell surface was exposed to air and culturing was continued for 10 days according to the manufacturer’s instructions. All experiments were independently performed in triplicate.

### Microarray gene expression and gene network analysis

A microarray gene expression analysis was performed using a GeneChip® system with a Human Genome U133-plus 2.0 array, which was spotted with 54,675 probe sets (Affymetrix, Inc., Santa Clara, CA, USA) according to the manufacturer’s instructions. Total RNA from three experiments was pooled, and 500 ng of the RNA was used to synthesize cRNA with a GeneChip® 3′IVT Expression Kit (Affymetrix, Inc.). The array was hybridized with biotin-labeled cRNA at 45 °C for 16 h. After treatment with phycoerythrin-labeled streptavidin, the array was scanned using a probe array scanner (Affymetrix GeneChip® Scanner 3000). The obtained hybridization intensity data were further analyzed using GeneSpring^®^ GX (Agilent Technologies, Inc.) to extract the significant genes and Ingenuity^®^ Pathway Analysis tools (Tomy Digital Biology, Co., Ltd., Tokyo, Japan) to examine gene ontology, including biological processes, cellular components, molecular functions, and gene networks^31,32^. The microarray data were deposited in the Gene Expression Omnibus: https://www.ncbi.nlm.nih.gov/geo/query/acc.cgi?acc=GSE136127.

### Cell growth and MTS assays

To measure cell growth rates, 5.0 × 10^4^ cells were plated onto 35-mm diameter plates. At each time point, cells were stained with trypan blue and were counted. For MTS assay, cells (1.0 × 10^3^/100 μl medium) were seeded in triplicate wells of 96-well plates. At each time point, cells were stained with 20 μl MTS dye (Promega CO, Fitchburg, WL) at 37 °C for 1 h. The absorbance was measured at 570 nm, with 655 nm as the reference wavelength. All experiments were independently performed three times.

### Western blotting

Protein extracts were prepared as described previously^33^. Ten milligrams equivalents of protein were applied onto 10% SDS–polyacrylamide gels (PAGEL; Atto, Tokyo, Japan), electrophoresed and transferred onto PolyScreen Transfer Membranes (NEN Life Science Products, Boston, MA). The membranes were treated with primary antibodies at 4 °C for 24 h, and positive signals were visualized using ECL prime Western Blotting Detection Reagents (GE Healthcare UK Ltd, Buckinghamshire, England).

### Immunostaining and TUNEL assays

Paraffin-embedded tissue sections obtained from human SCCs or skin-equivalent models were incubated with Dako target retrieval solution (DAKO) at a pH 9, at 121 °C for 4 min for the retrieval of antigens and subsequently blocked with Protein Block Serum-Free (DAKO) for 15 min. The sections were then incubated with primary antibodies for 60 min. Alexa Fluor 488 goat anti-rabbit IgG (H + L) or Alexa Fluor 555 goat anti-mouse IgG (H + L) (Molecular Probes, Eugene, OR) were used as secondary antibodies. 6-diamidine-2′-phenylindole dihydrochloride (DAPI) (Molecular Probes Inc.) was used for the visualization of nuclei. The TUNEL reaction was performed using a fluorescein in situ cell death detection kit (Roche Diagnostics Ltd.) according to the manufacturer’s instructions. The tissue sections were observed using a fluorescence microscope (Olympus). Cultured NHKs and HSC-1 cells were fixed with 4% paraformaldehyde; then, the cells were stained in the same manner.

The ratio of Ki67-positive cells or TUNEL-positive cells was calculated below. In a typical experiment, over 1500 cells in 10 fields were counted and the results were expressed as the ratio of positive cells. The experiments were independently repeated three times.

### The analysis of early apoptosis and secondary necrosis

Cells were collected 3 days after transfection with TCHHL1 siRNA or control siRNA and were processed using the annexin V-FITC kit (fluorescein isothiocyanate (FITC)-labeled annexin V/propidium iodide (PI) staining) according to the manufacturer’s protocol (Immunotech, Marseille, France). A total of 10,000 events were analyzed by flow cytometry (Epics XLTM; Beckman Coulter, Brea, CA).

### Statistical analysis

The values are expressed as the means ± standard deviation (SD). Three independent experiments were conducted for all assay conditions. Statistical significance was evaluated by the Mann–Whitney *U* test. *P* values of <0.05 were considered to indicate statistical significance.

## Supplementary information

Supplementary Figure S1

Supplementary Figure S2

Supplementary Figure S3

Supplementary Figure S4

Supplementary Figure S5

Supplementary Figure S6

Supplementary Figure S7

Supplementary Figure S8

Supplementary Figure S9

Supplementary Figure S10

Supplementary Figure S11

Supplementary Figure legends
