# Supplementary figures and images for: Trichohyalin-like 1 protein plays a crucial role in proliferation and anti-apoptosis of normal human keratinocytes and squamous cell carcinoma cells
Source: Cell Death Discov. 2020 Oct 27;6:109. doi: 10.1038/s41420-020-00344-5 (PMC7591909; doi:10.1038/s41420-020-00344-5)

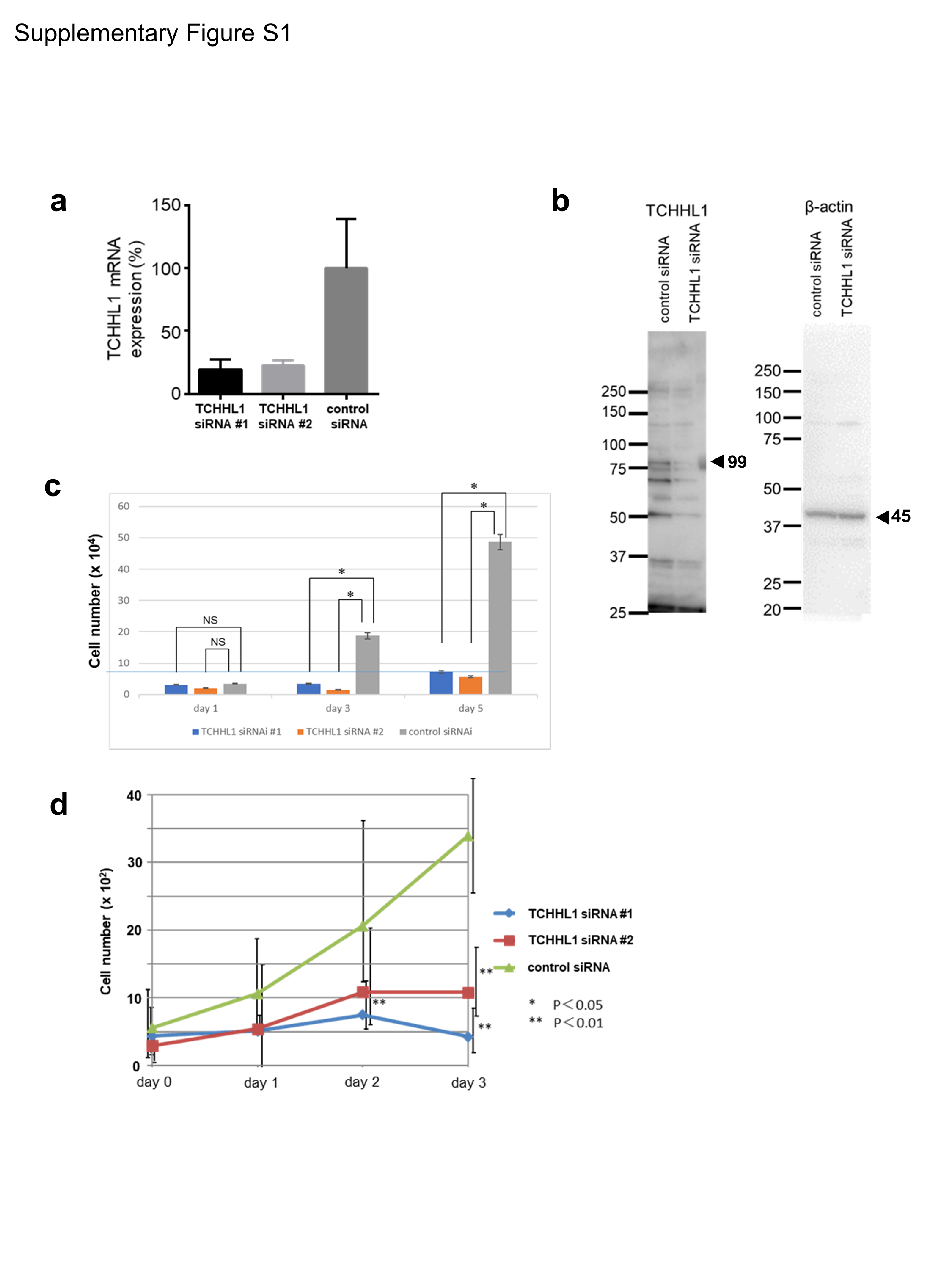

Supplement: Supplementary file 1 — Supplementary Figure S1 [file 41420_2020_344_MOESM1_ESM.tif]

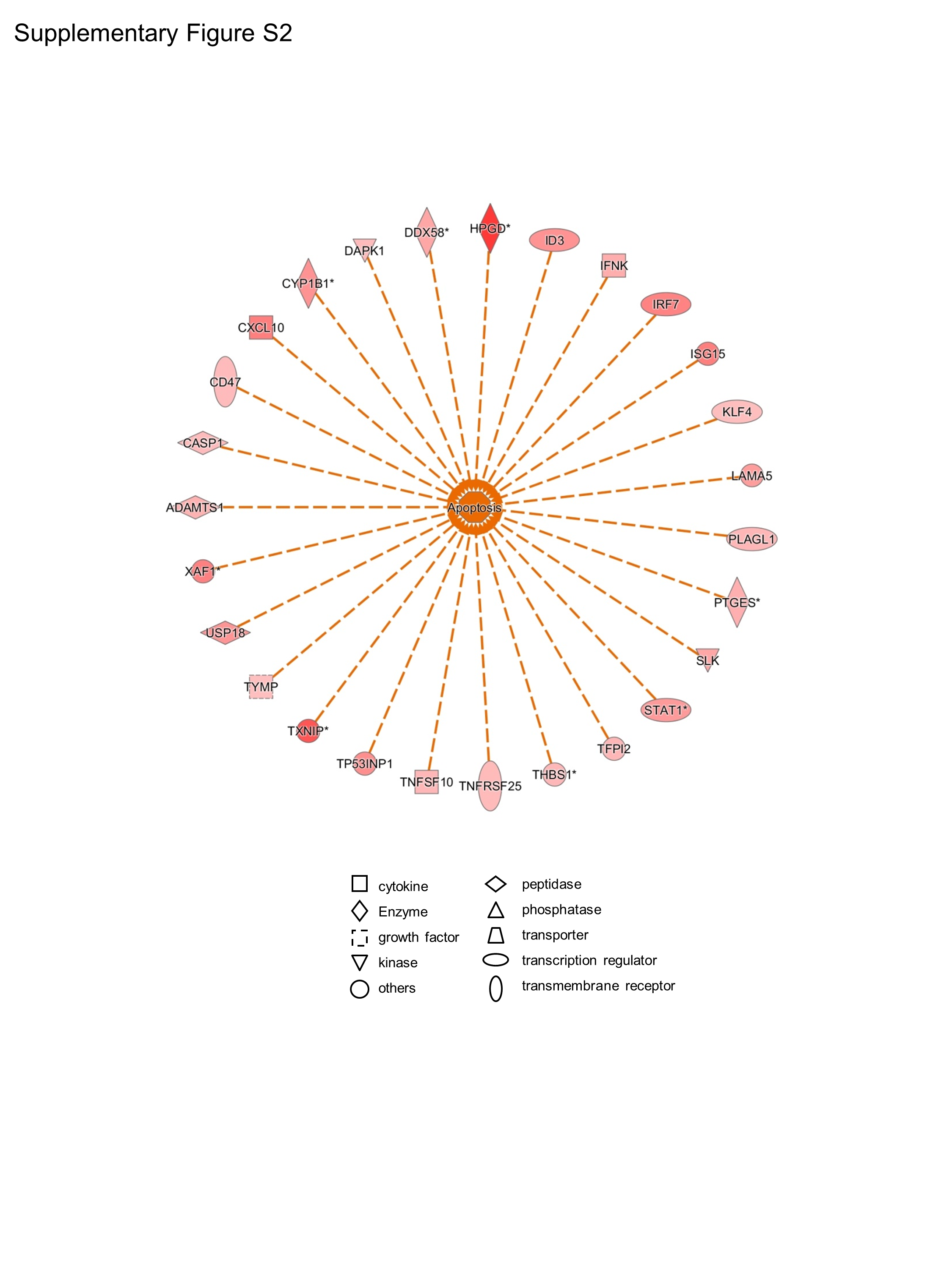

Supplement: Supplementary file 2 — Supplementary Figure S2 [file 41420_2020_344_MOESM2_ESM.tif]

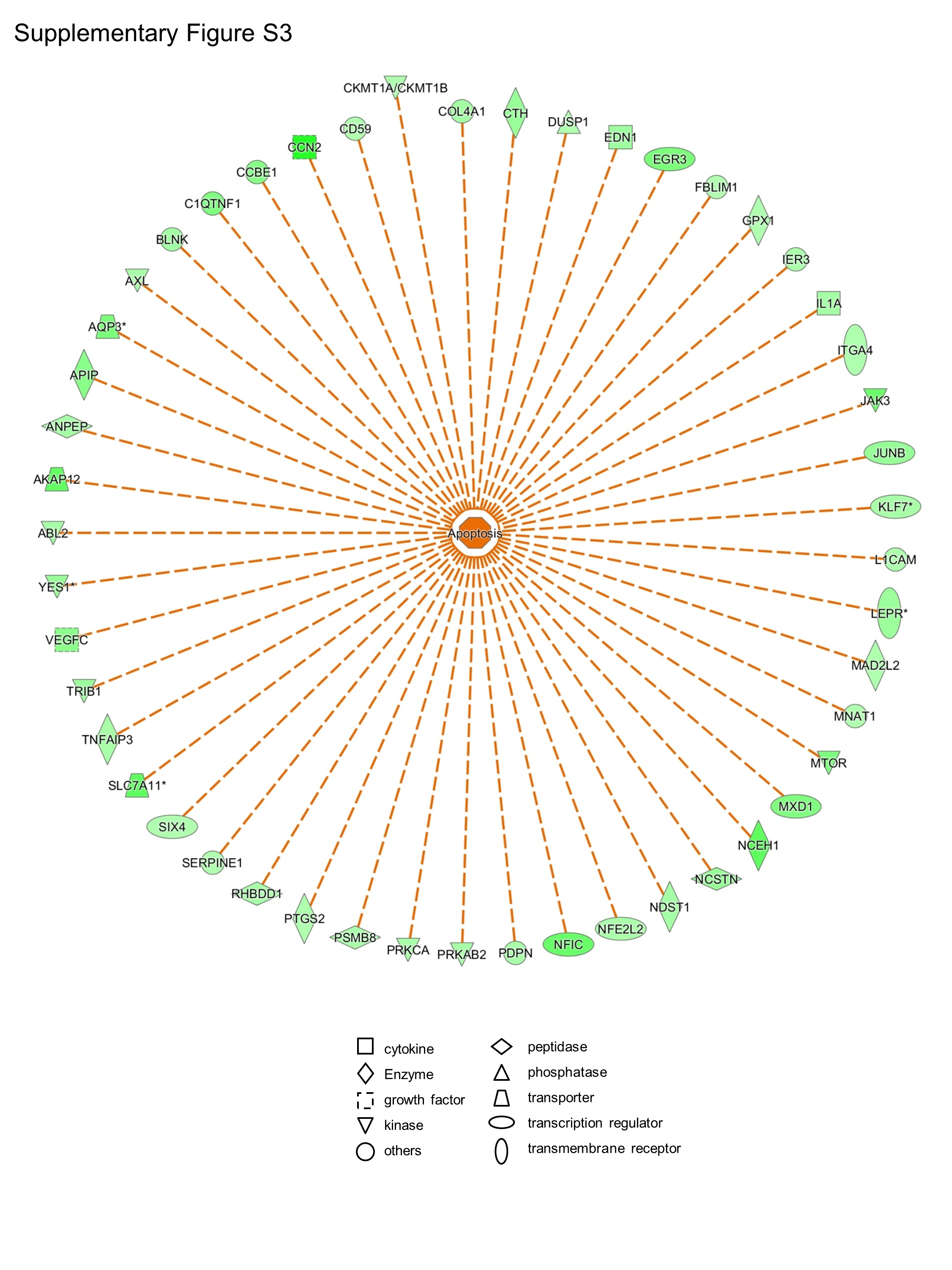

Supplement: Supplementary file 3 — Supplementary Figure S3 [file 41420_2020_344_MOESM3_ESM.tif]

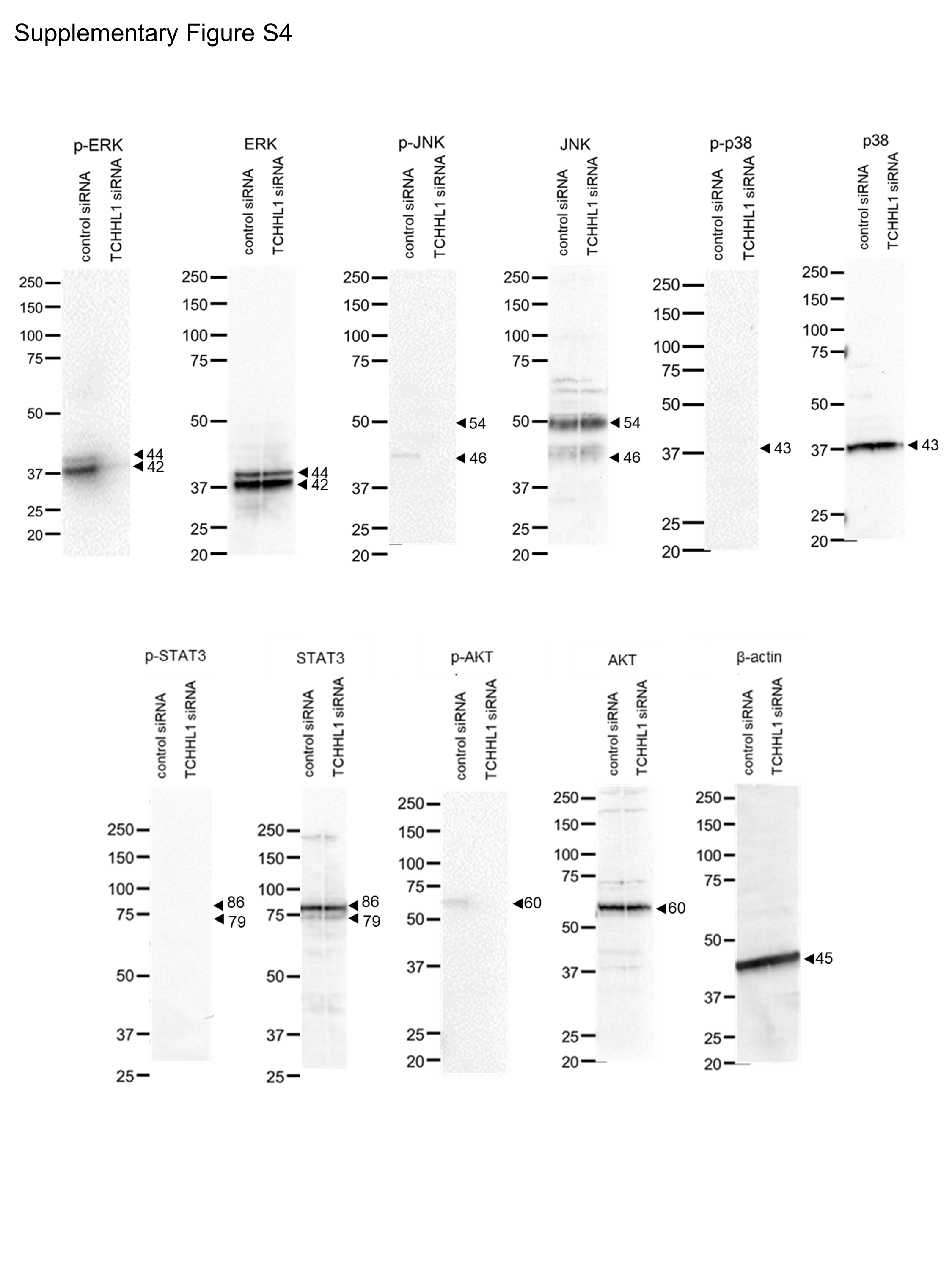

Supplement: Supplementary file 4 — Supplementary Figure S4 [file 41420_2020_344_MOESM4_ESM.tif]

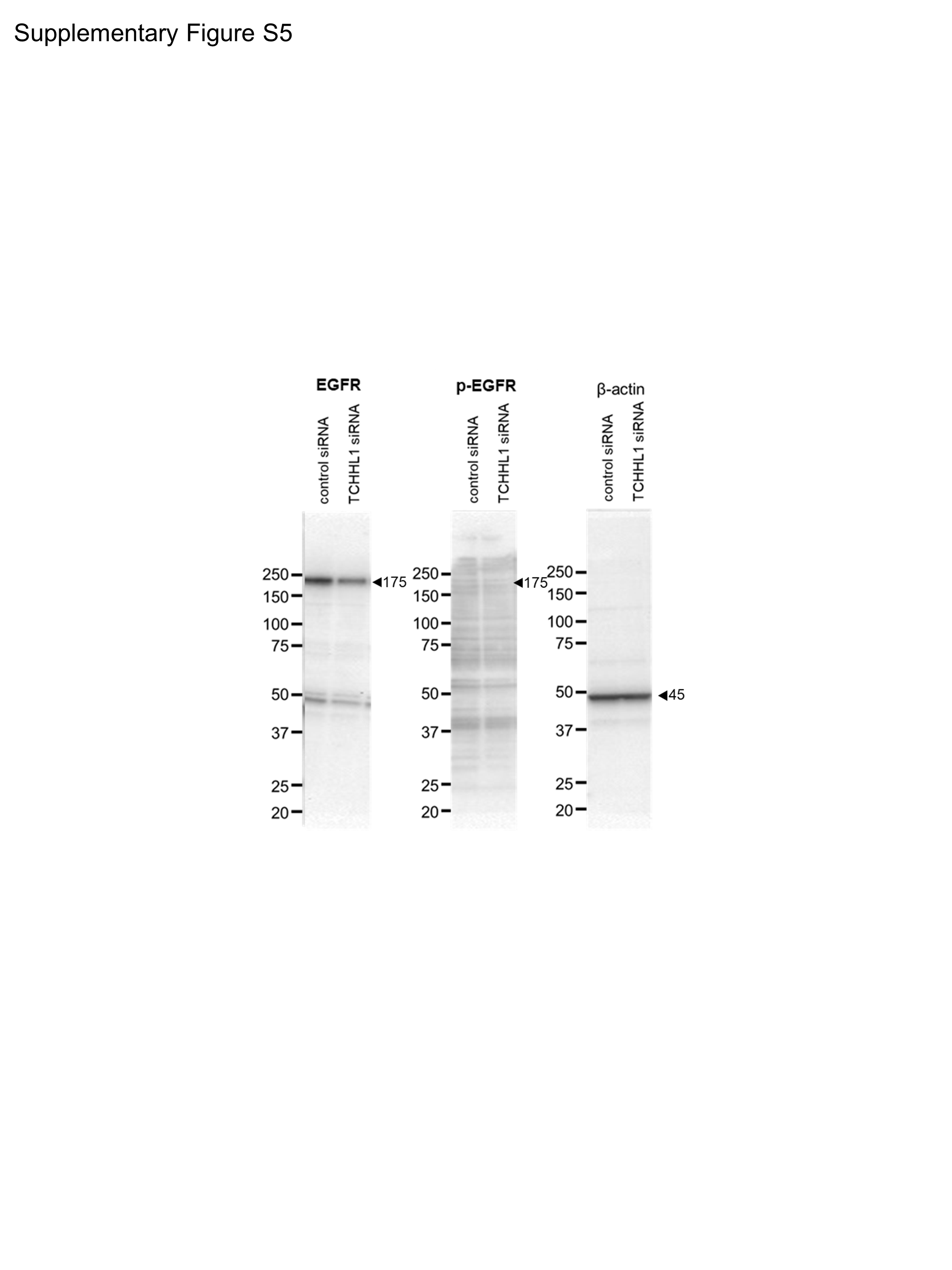

Supplement: Supplementary file 5 — Supplementary Figure S5 [file 41420_2020_344_MOESM5_ESM.tif]

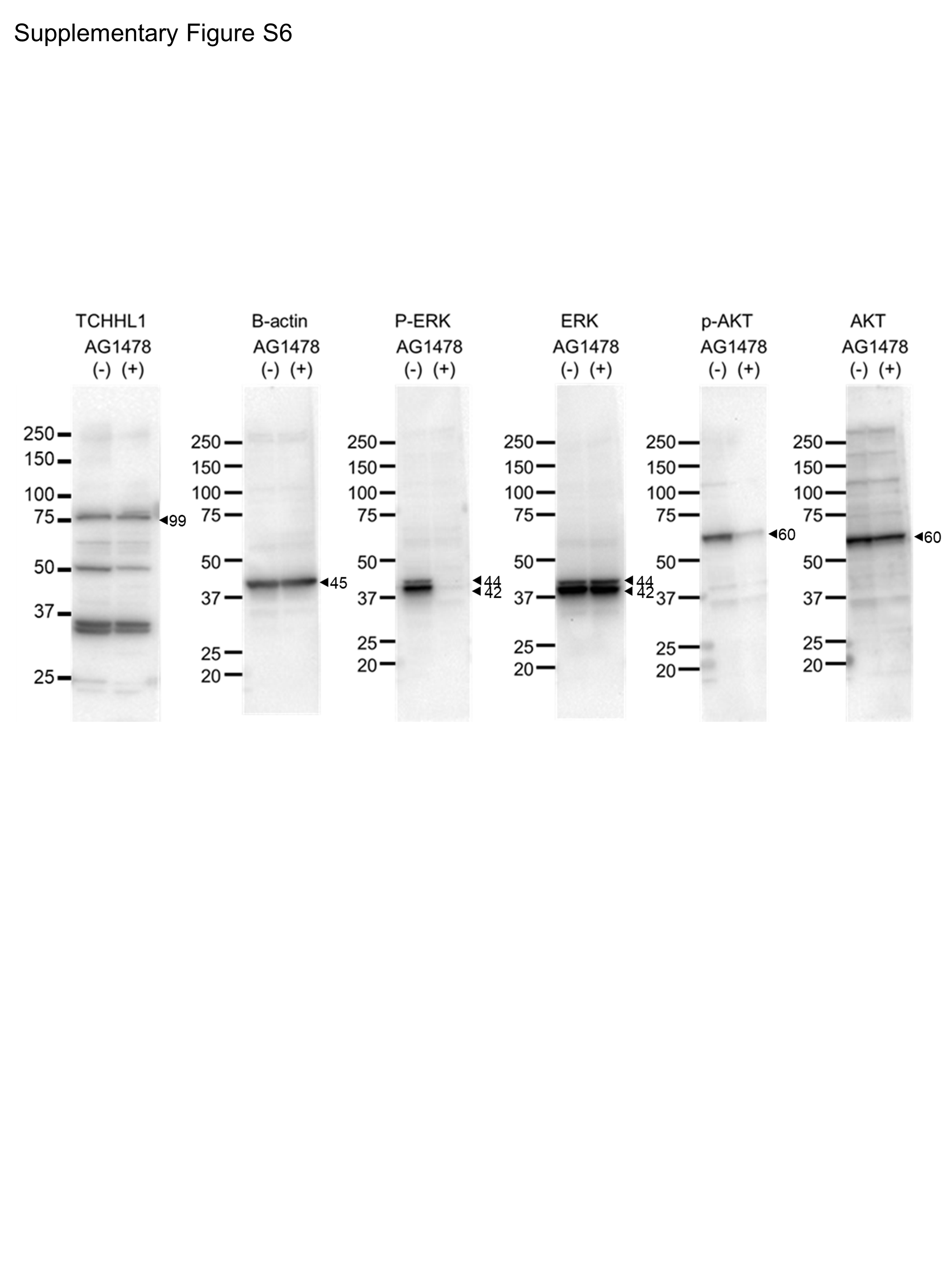

Supplement: Supplementary file 6 — Supplementary Figure S6 [file 41420_2020_344_MOESM6_ESM.tif]

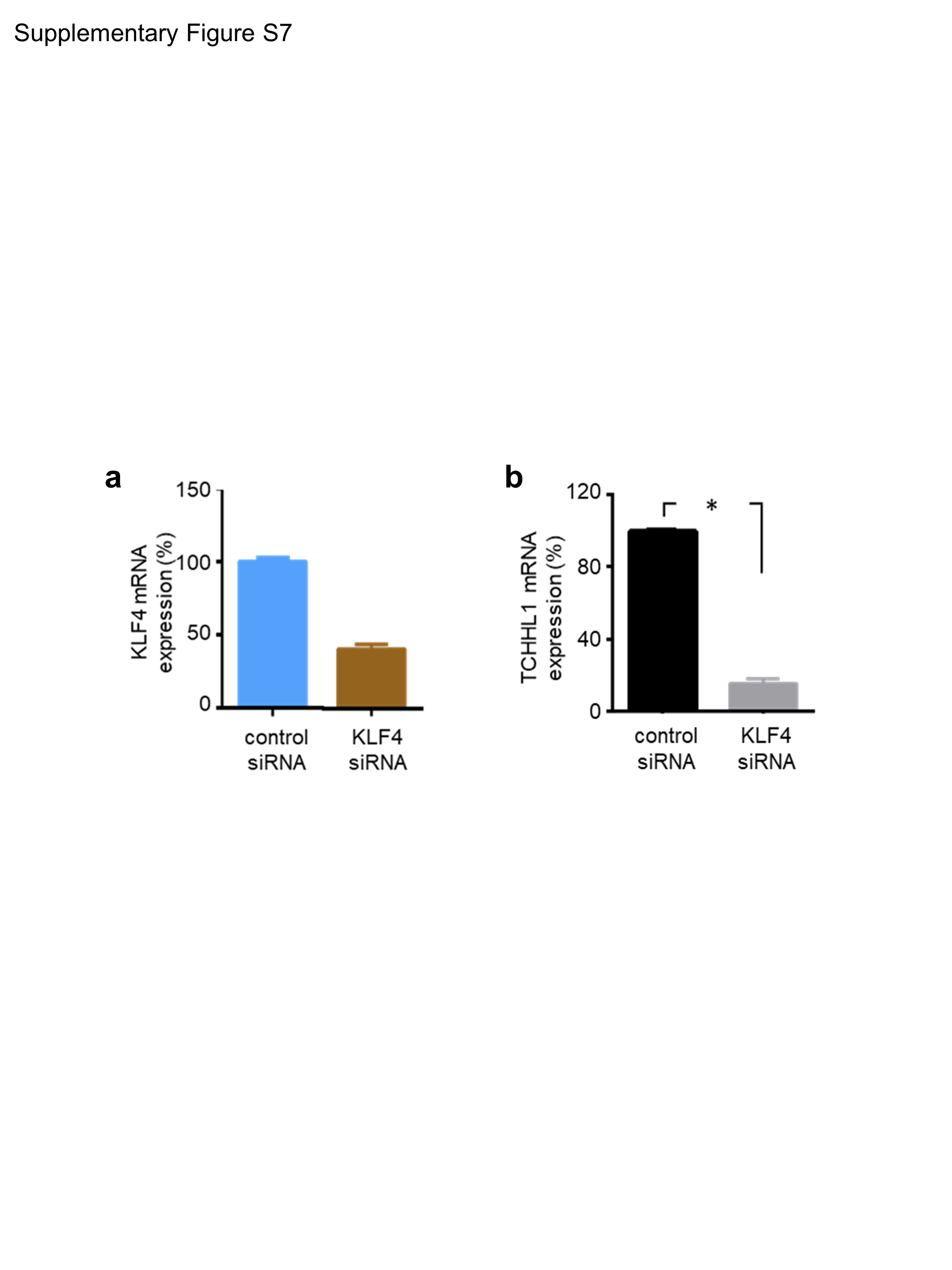

Supplement: Supplementary file 7 — Supplementary Figure S7 [file 41420_2020_344_MOESM7_ESM.tif]

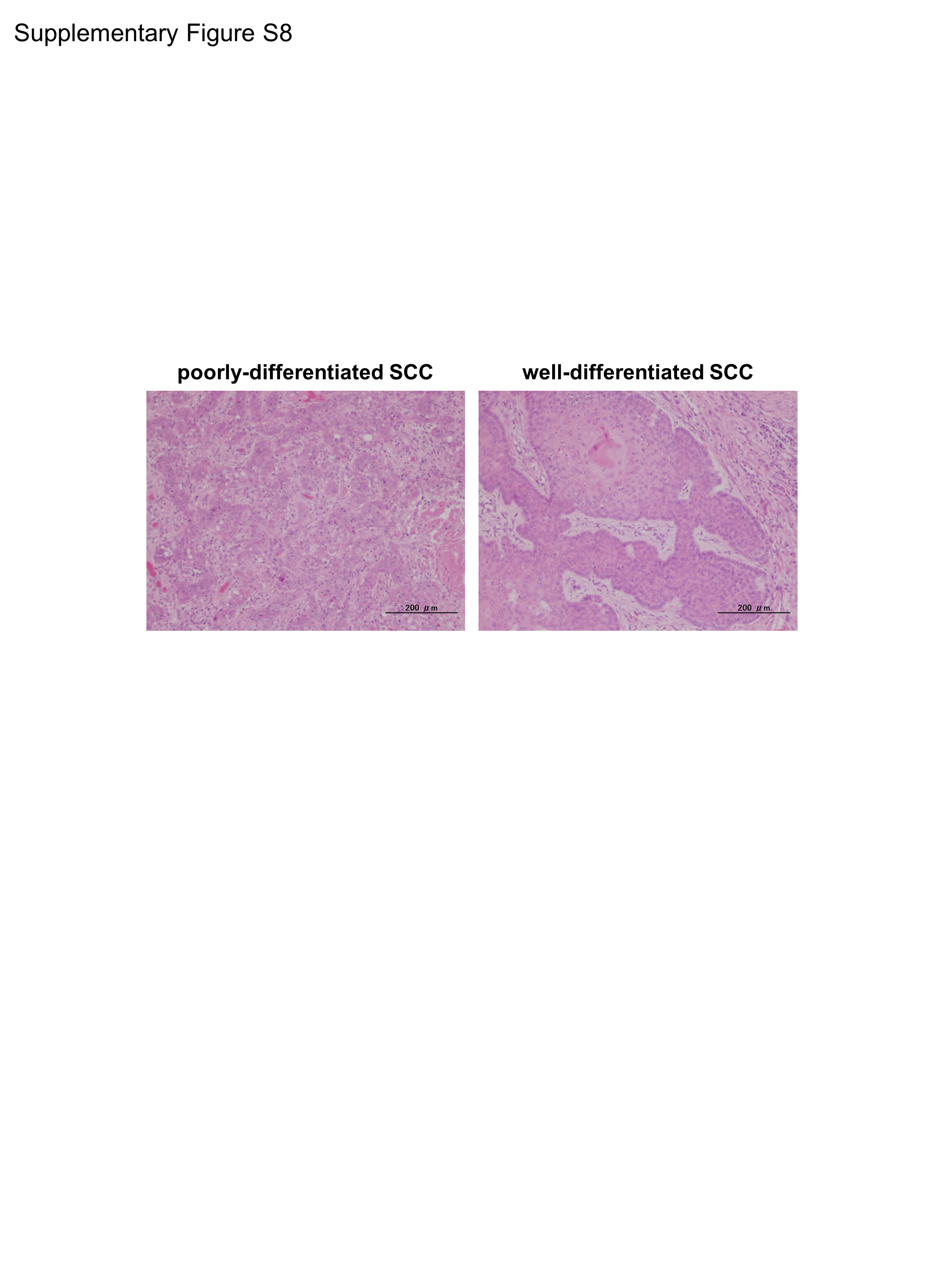

Supplement: Supplementary file 8 — Supplementary Figure S8 [file 41420_2020_344_MOESM8_ESM.tif]

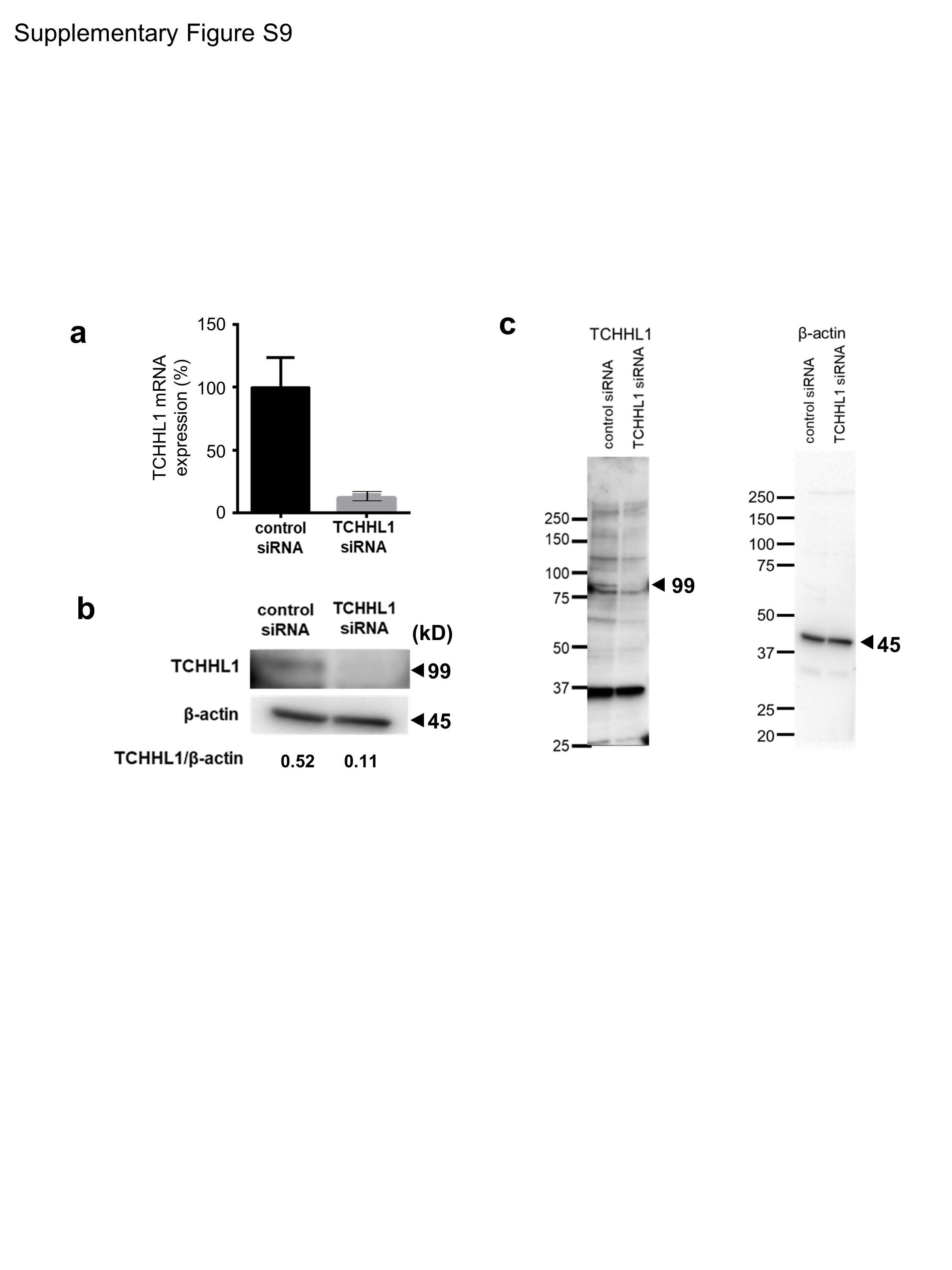

Supplement: Supplementary file 9 — Supplementary Figure S9 [file 41420_2020_344_MOESM9_ESM.tif]

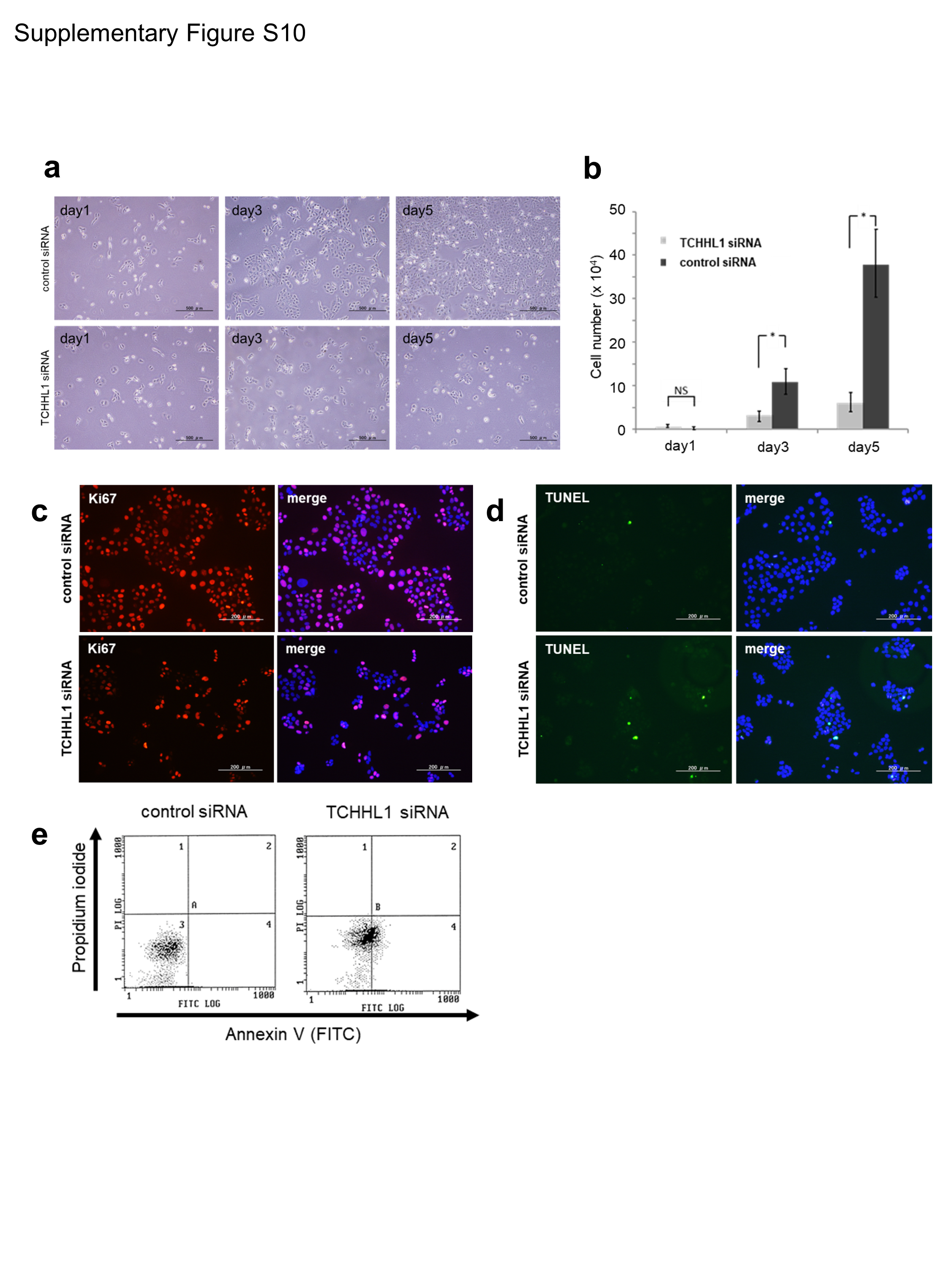

Supplement: Supplementary file 10 — Supplementary Figure S10 [file 41420_2020_344_MOESM10_ESM.tif]

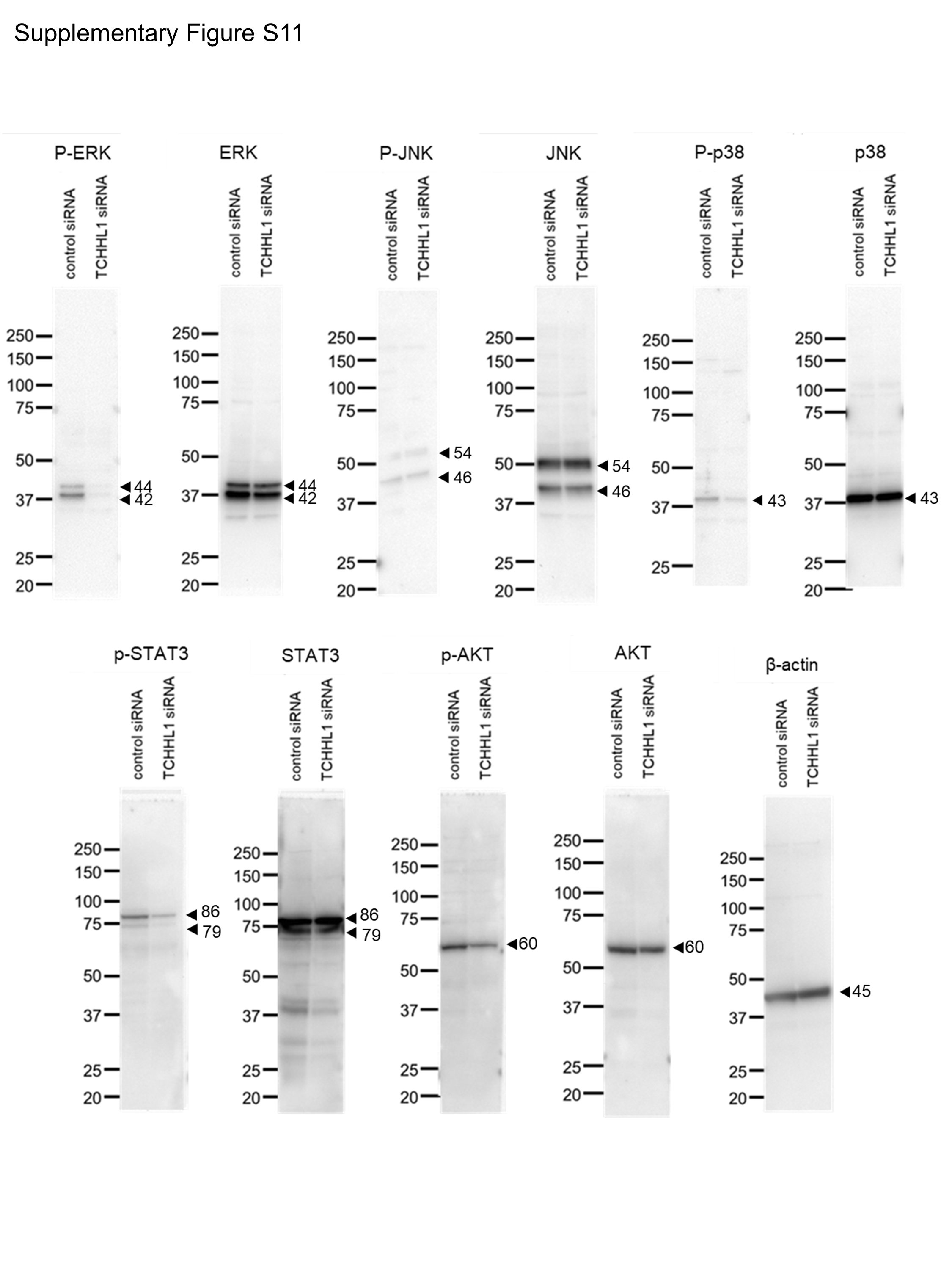

Supplement: Supplementary file 11 — Supplementary Figure S11 [file 41420_2020_344_MOESM11_ESM.tif]
