# Supplementary material for: Trichohyalin-like 1 protein plays a crucial role in proliferation and anti-apoptosis of normal human keratinocytes and squamous cell carcinoma cells
Source: Cell Death Discov. 2020 Oct 27;6:109. doi: 10.1038/s41420-020-00344-5 (PMC7591909; doi:10.1038/s41420-020-00344-5)
Supplement: Supplementary file 12 — Supplementary Figure legends [file 41420_2020_344_MOESM12_ESM.docx]

**Supplementary Figure legends**

**Supplementary Figure S1.** a) The TCHHL1 siRNAs strongly suppressed the expression of TCHHL1 mRNA. b) Full-length blots of Figure 1b. The TCHHL1 siRNAs suppressed the expression of TCHHL1 protein. c) Quantification of the number of growing NHKs at 1, 3, and 5 days after transfection with TCHHL1 siRNAs or control siRNA. The data represent the mean ± SD of three independent experiments. *p<0.05. d) Knockdown of TCHHL1 due to TCHHL1 siRNAs suppressed the growth rate of NHKs, as determined by MTS assay. The data represent the mean ± SD of three independent experiments. **p<0.01.

**Supplementary Figure S2.** Apoptosis-related upregulated genes. Upregulated genes were analyzed by the Ingenuity® Pathway Analysis tools. Many proapoptotic genes were upregulated, and apoptosis was predicted to be activated in the TCHHL1 siRNA-treated samples.

**Supplementary Figure S3.** Apoptosis-related downregulated genes. Downregulated genes were analyzed by the Ingenuity® Pathway Analysis tools. Many antiapoptotic genes were downregulated, and apoptosis was predicted to be activated in the TCHHL1 siRNA-treated samples.

**Supplementary Figure S4.** Full-length blots of Figure 3a. Western blotting of NHKs transfected with TCHHL1 siRNA or control siRNA. β-actin was used as a loading control.

**Supplementary Figure S5.** Full-length blots of Figure 3a. Western blotting of NHKs transfected with TCHHL1 siRNA or control siRNA. β-actin was used as a loading control.

**Supplementary Figure S6.** Full-length blots of Figure 3b. Western blotting of NHKs treated with AG1478 (EGFR inhibitor). β-actin was used as a loading control.

**Supplementary Figure S7.** a) Treatment with KLF4 siRNA in NHKs decreased the mRNA expression of KLF4 to 40.6% of the control NHKs. b) Relative mRNAs in NHKs with KLF4 siRNA or control siRNA were studied by quantitative reverse transcriptional PCR (RT-PCR), normalized to the β-actin value. The data represent the mean ± SD of three independent experiments. *p<0.01.

**Supplementary Figure S8.** The histological finding of cutaneous squamous cell carcinoma (Hematoxylin-Eosin staining). The poorly-differentiated SCC is histologically characterized by atypicality of tumor cells, keratinization of individual cells and the absence of an intercellular bridge. In contrast, the well-differentiated SCC is characterized by the presence of keratinization, which frequently takes place in the form of horn pearls.

**Supplementary Figure S9.** a) TCHHL1 siRNA strongly suppressed the expression of TCHHL1 mRNA to 17.1%. b) Western blotting showed a marked reduction of TCHHL1 protein (21.2%). C) Full-length blots of supplementary Figure 9b. Western blotting of HSC1 transfected with TCHHL1 siRNA or control siRNA. β-actin was used as a loading control.

**Supplementary Figure S10.** a) Representative micrographs of HSC1 cells at 1, 3, and 5 days after transfection of TCHHL1 siRNA or control siRNA. b) Quantification of the number of HSC-1 cells at 1, 3, and 5 days after transfection with TCHHL1 siRNA or control siRNA. The data represent the mean ± SD of three independent experiments. NS: not significant, *p<0.01. c) TCHHL1 siRNA or control siRNA were transfected into HSC1 cells that were stained using anti-Ki67 antibody (left panels). Nuclear staining by 6-diamidine-2’-phenylindole dihydrochloride (DAPI) appears in blue. Merged figures with nuclear staining are also shown (right panels). d) HSC1 cells transfected with TCHHL1 siRNA or control siRNA were subjected to TUNEL staining (left panels). Nuclear staining by DAPI appears in blue. Merged figures with nuclear staining are also shown (right panels). e) HSC-1 cells that were transfected with TCHHL1 siRNA or control siRNA were stained with annexin V and PI and assessed by a FACS analysis at 3 days after transfection of siRNAs.

**Supplementary Figure S11.** Full-length blots of Figure 7g. Western blotting of HSC1 cells transfected with TCHHL1 siRNA or control siRNA. β-actin was used as a loading control.
